# Evaluation of Phytoseiid and Iolinid Mites for Biological Control of the Tomato Russet Mite *Aculops lycopersici* (Acari: Eriophyidae)

**DOI:** 10.3390/insects13121146

**Published:** 2022-12-12

**Authors:** Juliette Pijnakker, Asli Hürriyet, Clément Petit, Dominiek Vangansbeke, Marcus V. A. Duarte, Yves Arijs, Rob Moerkens, Louis Sutter, Dylan Maret, Felix Wäckers

**Affiliations:** 1 R&D Department, Biobest Group N.V., 2260 Westerlo, Belgium; 2 Agroscope, Plant-Production Systems, 1964 Conthey, Switzerland

**Keywords:** Acari, Iolinidae, *Homeopronematus*, *Pronematus*, tomato, greenhouse, biological control, small iolinid mites, big impact on TRM

## Abstract

**Simple Summary:**

The tomato russet mite (TRM), *Aculops lycopersici* (Eriophyidae), causes severe damage to tomato plants *Lycopersicon esculentum,* which results in a wilted, russetted appearance with desiccated leaves. This study focused on the search for a suitable biological control agent against TRM, as an alternative to commonly used sulfur or chemicals. The efficacy of several potential predatory mite species was assessed. *Pronematus ubiquitus* proved successful in preventing the development of TRM and damage symptoms. The potential of iolinid predatory mites for the biological control of eriophyids is discussed.

**Abstract:**

Our search for a suitable biological agent to control the tomato russet mite (TRM), *Aculops lycopersici*, was initiated in 2013. *Neoseiulus californicus*, *Amblyseius andersoni,* and *Neoseiulus fallacis* showed a promising pest reduction potential in a curative control strategy. Although these beneficials had a low survival on tomato and were not able to eradicate the pest, plants did not present typical TRM damage. However, their inability to establish in the tomato crop means that their commercial use would require repeated introductions, making their use too expensive for growers. Other predatory mites in the survey, such as the iolinids *Homeopronematus anconai* and *Pronematus ubiquitus*, showed the potential for a preventative strategy as they can establish and reach high densities on tomato with weekly or biweekly provision of *Typha angustifolia* pollen as a food source. When the tomato crop was adequately colonized by either iolinid, the development of TRM and any damage symptoms could be successfully prevented. The potential of iolinid predatory mites for biological control of eriophyids is discussed.

## 1. Introduction

The tomato russet mite (TRM), *Aculops lycopersici* Massee (Eriophyidae), is a cosmopolitan pest of unknown geographical origin and original host [1]. The species is found in almost all agricultural regions where solanaceous crops are cultivated [1,2,3]. Due to the fact that TRM infestations usually remain undetected at the base of the tomato plant, the mite is able to progress towards the canopy [4]. The pest causes serious damage to the head of the tomato plants, resulting in a wilted, russetted appearance with desiccated leaves [5,6]. The abaxial side of the lower leaves is often silvered, and the stems lose trichomes and develop a brown color, often with small fissures. Fruits can become bronzed on highly infested plants. To avoid serious crop damage, tomato growers in northwestern Europe increasingly use preventative sulfur sprays (or evaporation) and chemical control measures with compounds such as abamectin and/or spiromesifen upon the first signs of plant damage (Juliette Pijnakker, personal experience). Since TRM is difficult to detect early-on and has a high reproduction capacity, with populations doubling in less than three days at 25 °C [7], the eradication of the pest is difficult.

Studies investigating the use of predatory phytoseiid mites against TRM (Duso et al., 2010) [6] are mostly limited to relatively small-scale laboratory experiments. Several phytoseiid predators have been observed feeding on TRM, including the phytoseiid mites *Amblyseius andersoni* (Chant) [8], *Amblydromalus limonicus* (Garman and McGregor) [9,10,11], *Amblyseius swirskii* (Athias-Henriot) [12,13,14], *Neoseiulus californicus* (McGregor) [8,15], *Neoseiulus cucumeris* Oudemans [8,16,17], *Neoseiulus fallacis* (Garman) [16], and *Typhlodromus* (*Anthoseius*) *recki* Wainstein [18]. Even though TRM is a suitable prey for some phytoseiids [16], their impact in terms of biological control is often insufficient [8,11,16]; therefore, phytoseiid mites are rarely used in commercial tomato crops against TRM. The capacity of phytoseiid mites to survive, move, and reproduce—and thus establish—on the tomato plants is hampered by glandular trichomes [11,14,19,20,21,22,23,24]. Furthermore, toxic secondary metabolites (in plants and prey) are thought to be lethal to the phytoseiids [24,25]. The poor performance of phytoseiids on tomato was confirmed in a few greenhouse trials on tomato plants in Europe. In France, *N. cucumeris* and *N. californicus* only reduced TRM populations when high numbers of predators were released: 3000 *N. californicus* and over 12,000 *N. cucumeris* per plant [17]. Although a single preventative release rate of 100 *A. andersoni* mites per plant) resulted in in a low TRM density on stems after eight weeks in comparison with a curative or simultaneous release of the predator, it could not fully eradicate the pest [8]. Curative releases of 140 and 420 *A. swirskii* individuals per tomato plant did not reduce the pest [11], and *A. limonicus* was also hampered by tomato trichomes [15].

Alternative options to promote the biological control of TRM include adapting predatory mites to the tomato plant [26] or developing tomato cultivars with fewer harmful trichomes [27,28]. Some mite species of the Tydeoidae, including the families Tydeidae and Iolinidae, are about five times smaller than most phytoseiid predators [29]. They are not hindered by the tomato trichomes, allowing them to move under and between the trichomes [30]. This applies to the mite species *Homeopronematus anconai* (Baker) [31], *Pronematus ubiquitus* (McGregor), and *Tydeus kochi* Oudemans [32,33,34,35]. Some of these species have been reported to occur naturally on tomato. The feeding habits of tydeids and iolinids range from predators, phytophages, mycophages, and parasitism on insects to scavengers [36,37]. Some species are reported to be pollen-feeders [31,38,39,40,41] and predators of small arthropods [16,31,42,43,44,45,46,47,48,49,50]. Many Tydeoidea species have been reported to be associated with eriophyoids, such as *H. anconai*, *P. ubiquitus*, *Pronematus staerki* Schruft [51], *T. kochi*, *Tydeus californicus* (Banks), *Tydeus caudatus* Dugès [52], *Tydeus caryae* Kanjani and Ueckermann, and *Tydeus goetzi* Schruft [53]. *Homeopronematus anconai* and *P. ubiquitus* are common species that have been well-studied in relatively small-scale laboratory experiments [30]. In 1961, Rice reported predation of TRM by *P. ubiquitus* [32]. Carmona found remnants of TRM in the gut of *P. ubiquitus* [33]. Hessein and Perring [31] found *H. anconai* in their TRM rearing and collected the predator from tomato plants. *Homeopronematus anconai* was able to develop and reproduce on TRM and succeeded in reducing TRM populations on tomato plants. Adults and nymphs of *H. anconai* and *P. ubiquitus* have been reported to kill all stages of TRM [30]. *Homeopronematus anconai* adults showed daily predation of about 70 *A. lycopersici* deutonymphs in the laboratory [54,55] or 3 to 4 adults of TRM adults per day [16]. Haque and Kawai [54,55] observed more than 2000 individuals of a naturally occurring population of *H. anconai* per leaf on tomato plants infested by *A. lycopersici*.

In this study, we investigated the efficacy of different predatory mites against TRM. The search for suitable biological agents for TRM started in 2013, when we compared the efficacy on individual plants of diverse phytoseiid predatory mites, either commercially available or from experimental rearing. Subsequently, following an important survey on Solanaceae, our focus shifted to iolinids, including a demonstration trial in a semi-commercial setting.

## 2. Materials and Methods

### 2.1. Pest, Beneficials, and Additional Food Source 

A population of *Aculops lycopersici* was maintained on potted tomato plants in a greenhouse of Biobest Group N.V. (Westerlo, Belgium) at 25 °C. Narrow-leaved cattail pollen (*Typha angustifolia* L., Nutrimite^™^), predatory mites, pest material, predatory bugs, and parasitic wasps to control whitefly populations during the trials were obtained from Biobest Group N.V.

### 2.2. Curative Releases with Nine Species of Phytoseiids on Individual Tomato Plants

To assess the efficacy of predatory mites in controlling TRM, a trial was conducted on individual tomato plants in a greenhouse of 150 m^2^ at the Greenlab facilities of Biobest Group N.V. The trial was performed from March to May 2013 at 70 ± 20% RH and an average temperature of 22 ± 8 °C with natural light. The plants were sown in the same facilities and grown in potted soil (Greenyard Horticulture, Ghent, Belgium) in 5 L pots. The pots were placed in containers on tables and watered by hand. A single head was kept per plant and all lateral shoots were removed weekly. No sulfur was used. *Encarsia formosa* Gahan was released preventatively against whiteflies.

Tomato plants cv. Marmande (Somers, Mechelen, Belgium) (1.5 month old, 6 leaves) grown in pots were placed on growing tables. Ten treatments (nine predator species tested alone and a control with only TRM) were assigned with three or six replicates using a randomized block design. For all treatments, one replicate was represented by one plant. Due to a shortage of breeding material, *Euseius ovalis* (Evans) and *Phytoseiulus macropilis* (Banks) were tested in three replicates only, whereas all other predatory mites treatments consisted of six replicates. 

On 25 March, a piece of tomato stem infested with approximately 500 *A. lycopersici* was attached with a thread between the 2nd and 3rd leaf. One day later, 500 mites of the species *A. limonicus*, *A. andersoni*, *A. swirskii*, *E. ovalis*, *Galendromus occidentalis* (Nesbitt), *N. californicus*, *N. fallacis*, and *P. macropilis* were released evenly over each plant. The predators were released each week for four consecutive weeks. All marketed predators were released in their commercial carrier, *E. ovalis* and *P. macropilis* were introduced in a sawdust carrier. *Amblyseius swirskii* was released either with or without cattail pollen as a supplemental food, while *E. ovalis* was always tested with pollen as Nutrimite^™^ is known to be particularly effective in boosting these two species [56]. The pollen (0.5 g/plant/week) was supplied weekly with a brush for four consecutive weeks after the predators’ introductions. Six weeks after the introduction of the pest and two weeks after the last release of the predatory mites, the number of healthy and damaged leaves and the percentage of stem russetted were assessed per plant to evaluate the efficacy of the predators. Eight leaves were removed per plant and examined under a stereomicroscope (Optika SZM-LED2, Ponteranica, Italy). The number of predators was counted per collected leaf to estimate the establishment of the predators. All predatory mites were collected at the end of the trials, to confirm species identity. The identification was done using a microscope (Zeiss Axio Scope.A1, Carl Zeiss, Jena, Germany) after mites had been mounted and cleared in a Marc André solution (Upton) [57] for three days on a warm plate.

All statistical analyses were performed using the statistical software R version 3.6.1 [58]. Differences in mite densities and the damage level on stem and leaf at the sampling period were analyzed with a general linear model using the function glht of the package lsmeans [59]. A pairwise post-hoc Tukey test was used to check for differences between objects. 

### 2.3. Preventative Releases of Two Iolinids Species with One Application of Pollen/Week on Individual Tomato Plants

In 2018, a trial was performed on individual tomato plants cv. Merlice (De Ruiter, Bergschenhoek, The Netherlands) to evaluate the impact of *H. anconai* and *P. ubiquitus* (supplied with cattail pollen) on TRM. Thirty-six plants were sown on 29 April and grown as described in the previous trial, except that the pots were placed in containers on the concrete floor, with sticky cardboard under each plant to avoid TRM contamination and movement of iolinids between treatments.

Treatments were arranged in a randomized block design with eleven replicates for the treatments with predatory mites supplied with pollen, and fourteen for the untreated control. For all treatments, one plant represented one replicate. The treatments were evaluated for 17 weeks from June until October in a greenhouse with natural light at 70 ± 20% RH and an average temperature of 21 ± 3 °C (setting: 19 °C night/20 °C day). Leaves, well-colonized by predators, were collected from a rearing on blackberry plants provided with the pollen of *T. angustifolia*. Leaves containing 50 predatory mites and about 10 eggs were introduced once preventatively on each tomato plant (of one stem) on 19 June. The berry leaves colonized with the predatory mites were placed on a tomato leaf against the stem at 90 cm from the growing substrate. The pollen (Nutrimite, Biobest Group N.V., 0.15 g per plant) was applied weekly with a pollen-blowing device (Nutrigun, Biobest Group N.V.). The control treatment received neither pollen nor predators. Plants were infested with the pest on 10 July, three weeks after the introduction of the predators. One infested tomato stem piece with ca. 300 TRM (mixed stages) was fixed with a wire to the tomato stem next to the release point of the predatory mites.

To follow the population dynamics of TRM and predatory mites, nine (3 and 5 weeks after predator release) and twenty-seven leaflets (9, 13, and 17 weeks after predator release) were collected from each plant in three strata (top, middle, bottom) of the plant. The number of tydeoids and eriophyoids was counted per leaflet using a stereomicroscope (Optika SZM-LED2). Microscopic slides of 60 predatory mites were made per plant to confirm the predators’ identity. The first damage was only visible 5 weeks after the release of the pest. The number of green, yellow, and bronzed leaves was first counted weekly from 14 August to 28 August and then biweekly until 19 October (16 weeks after the release of the predator and 13 weeks after the release of the pest). 

The number of predatory and pest mites and green leaves was analyzed among treatments with a linear mixed-effects model (LME) with treatment and time as fixed factors and plant identity as a random factor to correct for repeated measures [58]. 

### 2.4. Demonstration Trial in Semi-Commercial Conditions

In 2021, a demonstration trial was organized in an experimental crop with tomato plants cv. Marinice (De Ruiter, The Netherlands) to demonstrate the control of TRM by *P. ubiquitus* supplied with cattail pollen, compared with untreated plants without iolinids. *P. ubiquitus* was selected as it reproduces faster on pollen than *H. anconai* [30]. 

In total, 240 plants were sown on 28 April in a nursery and grown in rockwool slabs in a greenhouse of 180 m^2^ from 16 June to 6 October. Temperatures averaged 20 °C with a range of 16.5–21.5 °C. The relative humidity was 75 ± 10% RH. Two heads were kept per plant and all auxiliary shoots were removed weekly. The lowest leaves were cut off once the plants reached a total number of 18 leaf branches. This procedure was continued during the trial in order to assure that the total number of leaf branches did not exceed 18. The predatory bug *Macrolophus pygmaeus* Rambur (2 × 1/m^2^ on 20 and 27 July) and *Encarsia formosa* Gahan (12.5/m^2^/week) were released to control whiteflies. *Phytoseiulus persimilis* Athias-Henriot (25/m^2^/week + 250/m^2^ on 28 July, 4, 11 and 18 August), *Feltiella acarisuga* (Vallot) (10/m^2^/week on 28 July, 4, 11 and 18 August), and *Diglyphus isaea* Walker (2 × 5/m^2^ on 1 and 8 September) were introduced to control spider mites and leaf miners. 

Half of the greenhouse was inoculated with *P. ubiquitus*. Two rows of gutters were kept empty to separate the two sections of the greenhouse. There was no extra physical barrier to prevent the spread of mites between the treatments. Per plant (two stems), 100 mixed stages of the mites were introduced in a sawdust carrier on the top of the plants on 16 June and once more on 1 July. Four weeks after the last introduction of the predators, eight plants per treatment were infested with TRM on 28 July, and later two more infestations on 26 August and 2 September to speed up infection. An infested tomato stem piece with respectively ca. 50, 50, and 500 mixed stages of TRM was therefore fixed to the tomato stems below the bottom leaf of each plant. 

Initially, pollen (500 g/ha) was blown weekly until 7 August (7 weeks after the first release of the predatory mites) over the plants and then biweekly with the Nutrigun until the end of the demonstration. The control treatment received neither pollen nor predators. 

The development of the predator and the pest was followed until 6 October. From 7 July (third week after the first release of *P. ubiquitus*), twenty-five leaflets were collected weekly randomly in three strata (top, middle, down) of the plant in each treatment. To assess TRM, six leaflets were collected from three leaves of each infested plant. The TRM infestation level on stems was evaluated with two stickers placed around the stem (9.5 cm long) nearby the release point of the pest, with the sticky part towards the plant. All leaflets and stickers were examined under a stereomicroscope to count the number of predatory mites and eriophyoids. At the end of the demonstration, the number of plants presenting TRM damage was counted and the stems of the infested plants were examined for brown symptoms. The number of pest mites was analyzed with a linear mixed-effects model (LME).

## 3. Results

### 3.1. Curative Releases with Nine Species of Phytoseiids (Individual Plants)

**Figure 1:**
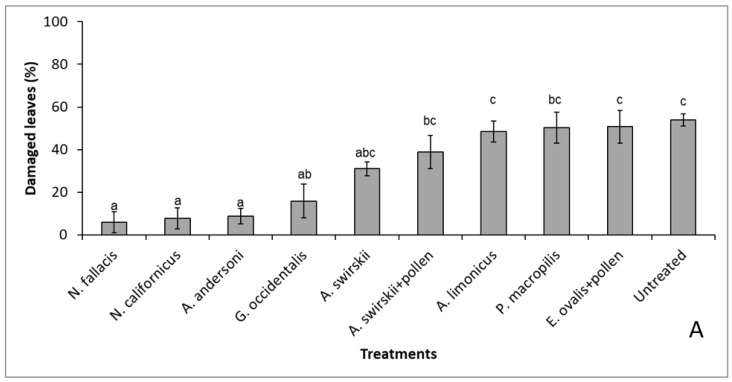

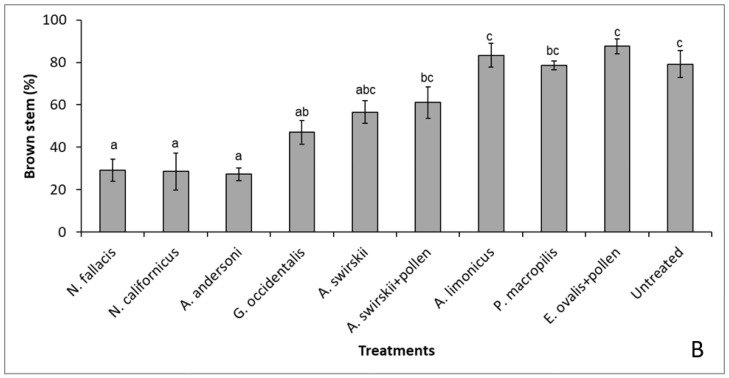
Percentage of damaged leaves (**A**) and brown stem (**B**) (mean ± SE) caused by TRM on the tomato plants of the different treatments six weeks after TRM inoculation and following four weekly curative releases of 500 predators per plant. Different letters above the data points denote significant differences among treatments after the generalized linear model (*p* < 0.05).

**Figure 2:**
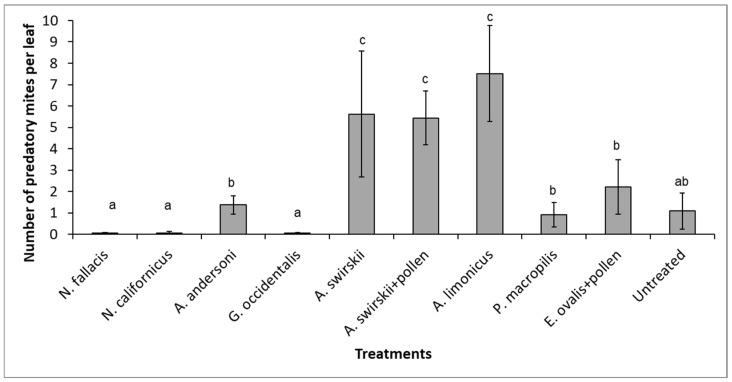
Number of established predatory mites (mean ± SE) on tomato plants infested with TRM six weeks after TRM inoculation and following four weekly curative releases of 500 predators per plant (*A. limonicus* and *A. swirskii* found on untreated plants). Different letters above the data points denote significant differences among treatments after the generalized linear model (*p* < 0.05).

*Neoseiulus californicus*, *A. andersoni,* and *N. fallacis* showed the most pronounced TRM damage reduction. There was a significant interaction between the treatments and the damage level of the leaves and the stems (Figure 1A,B). Although these species had a low survival (Figure 2) and the curative strategy did not eliminate the pest, plants remained healthy (Figure 1). Only *A. swirskii* and *A. limonicus* developed and reproduced well (Figure 2). 

### 3.2. Preventative Releases of Homeopronematus Anconai and Pronematus Ubiquitus with One Application of Pollen/Week (Individual Plants)

**Figure 3:**
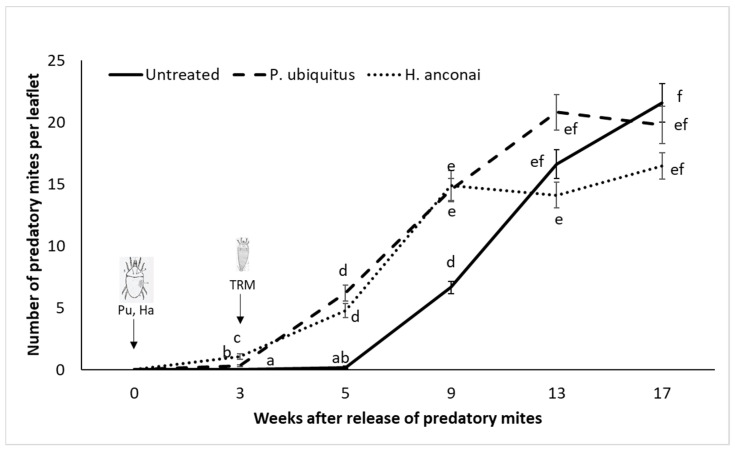
Population dynamics of predatory mites (Pu *Pronematus ubiquitus* and Ha *Homeopronematus anconai*) on tomato plants for 17 weeks. Different letters indicate a significant difference in the number of iolinids between the inoculated tomato plants and the control plants (linear mixed-effects models, *p* < 0.05).

**Figure 4:**
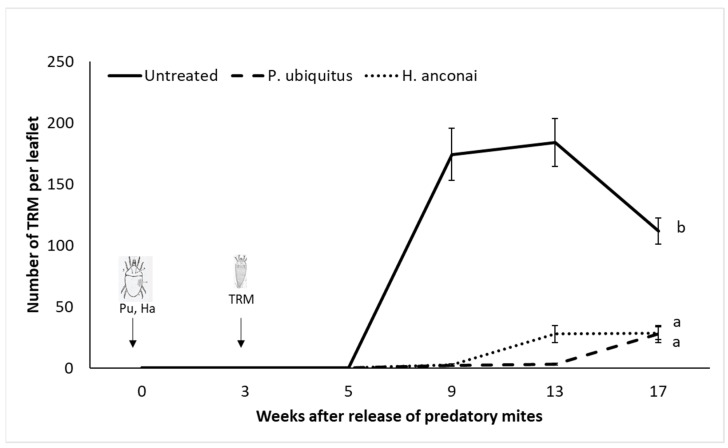
Population dynamics of TRM on tomato plants for 14 weeks. Different letters indicate a significant difference in the number of *Aculops lycopersici* between the inoculated tomato plants and the control plants (linear mixed-effects models, *p* < 0.05).

Both iolinids reached a density of about five mites per leaflet five weeks after their first introduction (Figure 3). Their populations increased to 15–20 predatory mites per leaflet nine weeks after their release. Both iolinids species were also found in week nine on the control plants, which were highly infested by the pest (Figure 4). 

**Figure 5:**
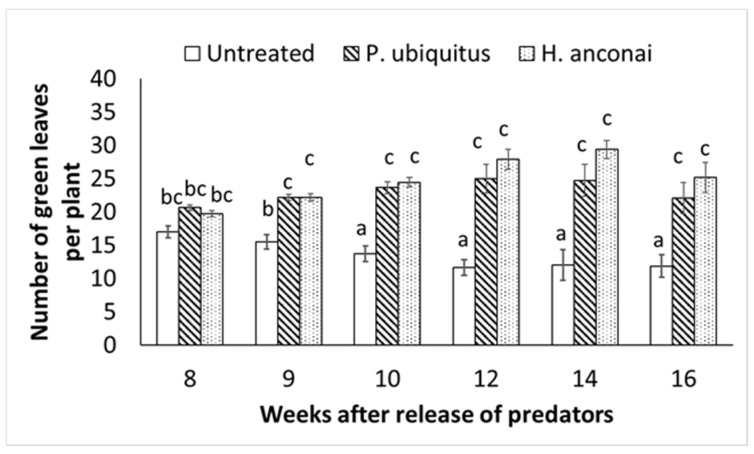
Remaining healthy (green) leaves per treatment. Different letters indicate a significant difference in the number of green leaves between the inoculated tomato plants and the control plants (linear mixed-effects models, *p* < 0.05).

Plants where the predators established at an early stage presented drastically fewer TRMs and less damage (Figure 4 and Figure 5). Damage was limited to the area of the inoculation point of the pest. 

### 3.3. Demonstration Trial in Semi-Commercial Conditions

**Figure 6:**
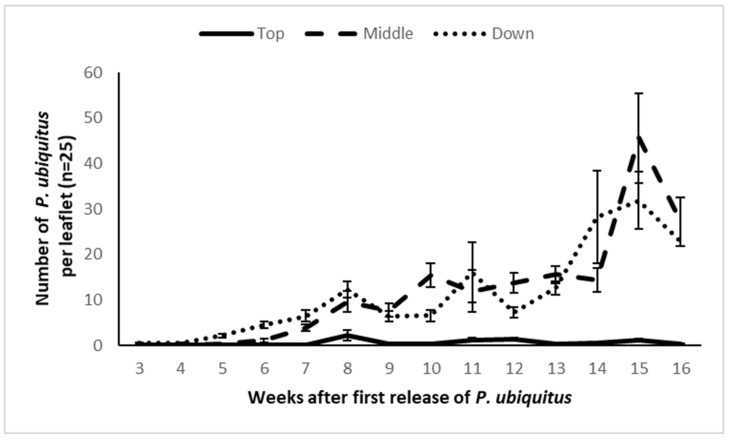
Population dynamics of *Pronematus ubiquitus* (mean number of mobile mites per leaflet ± SE) in an experimental greenhouse tomato crop.

**Figure 7:**
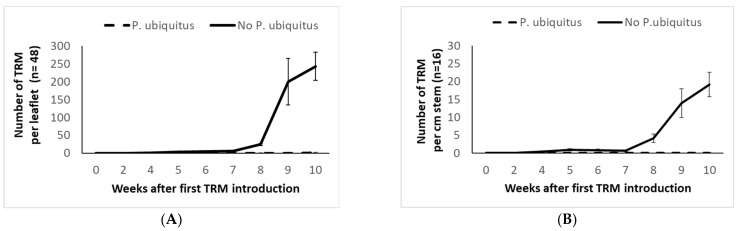
Population dynamics of TRM (mean number of mobile mites ± SE) found on the leaflets (**A**) and on the stem (**B**) nearby the point of the artificial infestation in an experimental greenhouse tomato crop.

Six weeks after the first release, the predator reached a density of five predators per leaflet (Figure 6). Only the top leaves showed a low predatory mite density (Figure 6). Two weeks after the inoculation of 500 TRM per stem, the pest population on the control plants increased drastically, both on the leaves (Figure 7A) and the stems (Figure 7B). At the end of the demonstration, 38.7 ± 4.9% (mean ± SE) of the artificially infested stems became rusty brown in the untreated plot, and the pest spread beyond the inoculation plants. Sixty-six stems out of the 240 stems without *P. ubiquitus* presented leaves with TRM damage. Plants which had been colonized by *P. ubiquitus* remained damage-free throughout the trial (green stem and no damaged leaves). Only a few living pest mites were recorded in the *Pronematus* plots (average of 10.2 ± 8.4 TRM per leaflet on 10.4% of the examined leaflets).

## 4. Discussion

Many phytoseiid species feed on TRM as prey in laboratory studies [16]. However, most of these predators are not effective biocontrol agents of TRM in greenhouse tomatoes as their establishment is hampered by the plant trichomes [10,24]. In our trial, only *A. swirskii* and *A. limonicus* became established, and only on damaged leaves with collapsed trichomes. Our studies confirmed that *N. californicus* [17], *A. andersoni* [8], and *N. fallacis* [16] could be used in an inundative strategy against TRM. However, as these phytoseiid mites do not establish on tomato plants, the high numbers and repeated introductions required are not economically viable for growers or could only be applied locally where TRM infestations are first expected (along the paths, the façades, and heating pipes). 

Unlike the tested phytoseiid species, the iolinid species *H. anconai* and *P. ubiquitus* showed a strong affinity for tomato crops, as already reported by Van Houten et al. [60] and Pijnakker et al. [61]. These tiny mites (225–280 µm) can survive on healthy tomato plants and circulate under and between the plant trichomes. In commercial tomato crops, these tydeoids are sometimes observed to occur spontaneously, probably feeding on tomato pollen, fungi in the phyllosphere, tarsonemids, and/or spider mites [62,63,64]. In our trials, both predatory mites reached high densities when released preventatively and supplied weekly with cattail pollen. A five or six week period was often needed to observe a density of about five predators per leaflet on tomato plant when only pollen was available. Most predatory mites were found in the bottom and middle strata, and less often in the top. Average densities of 20 predatory mites per tomato leaflet (top, middle, low leaves) were common in our controlled experimental designs, with numbers higher than 100 mites per leaflet at best. In contrast, most phytoseiid predators failed to establish or had populations 25 times smaller than the iolinid predators. Previously, Hessein and Perring [40] found a 4-fold increase in the survival of *H. anconai* (10–39%) when they added pollen to a TRM diet. Duarte et al. [62] showed that *P. ubiquitus* was able to reproduce on tomato pollen as a sole food source, but that *T. angustifolia* pollen yielded a higher reproduction than tomato pollen. As for reproduction on TRM alone, Vervaet et al. [30] found an oviposition rate over five days of 14.5 ± 1.7 and 14.4 ± 1.4 eggs for *P. ubiquitus* and *H. anconai*, respectively. Both species could reproduce and survive on diet of solely TRM. However, the predators’ fecundity reported by Vervaet et al. [30] was increased 1.7-fold when cattail pollen was added to the diet of TRM, with *P. ubiquitus* reproducing faster than *H. anconai*.

Brodeur et al. [16] considered *H. anconai* unsuitable for biological control of TRM in tomato crops, questioning the requirement of the predator for relatively high temperatures (28–30 °C optimal) and pollen for the oogenesis [65]. Our study demonstrated the preventative biological control potential of both iolinids with the addition of pollen on tomato plants at field realistic temperatures. Vervaet et al. [30] found that the presence of pollen lowers the predation of TRM by both iolinid species in laboratory trials. However, this does not need to compromise biocontrol at the plant level, as increased reproduction more than compensates for the reduced predation of individual mites [66]. Our study showed that both species could reduce TRM population densities on tomato plants when established prior to the inoculation of the pest. For *H. anconai,* these results are consistent with those of Hessein and Perring [31], where the predator reduced the pest infestation by 94.7% and 98.31%, respectively, one and two weeks after the first predatory mites were noticed. In our demonstration trial, the tomato plants showing a high colonization by *P. ubiquitus* were protected from *A. lycopersici* and outbreaks of the pest were avoided. This confirms the results obtained in a trial on individual tomato plants [63]. In our second trial, the decrease in TRM numbers found on the control plants in week 17 are also likely explained by the contamination of the control plants with a high number of iolinids.

Although the direct predation effect of both iolinid species on TRM has been well-studied [30], the elicitation of plant defensive secondary metabolites by the iolinids can be a further mechanism underlying the effects, as both predators complement their diets with plant feeding. These indirect effects are already widely discussed for other plant-feeding organisms [67,68,69]. Importantly, we have never observed any visible damage nor negative impacts on plant growth, even at the high densities obtained in our trials. 

Iolinids have not been used as biological control agents until now as pollen or TRM are needed to obtain effective high densities of predators [38,39]. Furthermore, the mass-rearing of the predators is complicated by the fact that plant tissue seems required for their survival [31,40,64]. However, since cattail pollen and other food supplements have been made available as commercial products, supplementing food has now become common practice in biocontrol programs in several greenhouse crops [70,71,72]. The new commercial availability of cattail pollen also allows the implementation of iolinids as predators of TRM. Pollen has to be supplied at least biweekly for a proper establishment of the iolinids [62]. A suitable release strategy will have to be elaborated, taking into account many aspects such as the moment and method of introduction, the dose, frequency and application method of pollen, the moment and importance of deleafing of the tomato plants, the presence of initial chemical residues, and compatibility with the use of fertilizers, (bio)pesticides [73], and other beneficials. The use of sulfur, as it is currently used against TRM as well as against powdery mildew, impedes the use of iolinids in greenhouse crops. The establishment of *P. ubiquitus* was considered to be impossible, due to the central role of sulfur [60]. However, recent studies have shown that the iolinids not only control TRM, but also effectively suppress powdery mildew [63], opening up opportunities for sulfur-free tomato production. 

## 5. Conclusions

In summary, we showed that *N. californicus*, *A. andersoni,* and *N. fallacis* are effective predators of TRM when released repeatedly in high numbers. Still, they cannot maintain populations on healthy or lightly infested plants as they are impeded by tomato trichomes. 

We confirmed that *H. anconai* and *P. ubiquitus* are extremely suitable predators of TRM in tomato. The predatory mites can survive and reproduce on tomato plants when they are provided with pollen. Through preventative introductions and (bi)weekly pollen applications, the iolinid predators can reach high population densities. The high predator densities keep TRM populations low. Our study reinforces the importance of preventative establishment and ‘power by numbers’ in biocontrol.

## Data Availability

Not applicable.
